# Factors influencing the adoption, implementation, and continuation of physical activity interventions in primary health care: a Delphi study

**DOI:** 10.1186/1471-2296-14-142

**Published:** 2013-09-26

**Authors:** Johanna M Huijg, Mathilde R Crone, Marieke W Verheijden, Nicolette van der Zouwe, Barend JC Middelkoop, Winifred A Gebhardt

**Affiliations:** 1 Clinical, Health and Neuropsychology, Leiden University, Wassenaarseweg 52, Leiden, the Netherlands; 2 Department of Public Health and Primary Care, Leiden University Medical Center, Hippocratespad 21, Leiden, the Netherlands; 3 TNO, Wassenaarseweg 56, Leiden, the Netherlands; 4 Regional Public Health Service Hollands Midden, Parmentierweg 49, Leiden, the Netherlands

**Keywords:** Physical activity, Interventions, Primary health care, Introduction, Adoption, Implementation, Continuation, Delphi, Importance, Changeability

## Abstract

**Background:**

The introduction of efficacious physical activity interventions in primary health care is a complex process. Understanding factors influencing the process can enhance the development of effective introduction strategies. This Delphi study aimed to identify factors most relevant for the adoption, implementation, and continuation of physical activity interventions in primary health care by examining experts’ opinions on the importance and changeability of factors previously identified as potentially relevant for the process.

**Methods:**

In the first round, 44 experts scored factors on their importance for each stage of the introduction process, as well as on their changeability. In the second round, the same experts received a questionnaire containing a reduced list of factors, based on the first-round results. They were asked to indicate their top-10 most important factors for each stage, and to re-rate factors’ changeability. Thirty-seven experts completed this round.

**Results:**

Most important factors could be identified for each stage. Some factors were found important for a specific stage, e.g., the presence of intervention champions within the organization (adoption), provider knowledge (implementation), and the intervention’s sustainability (continuation), while others were perceived important for all stages, i.e., the intervention’s financial feasibility, the intervention’s accessibility to the target group, and time to deliver the intervention. The majority of most important factors was perceived changeable. However, for some factors no consensus could be reached regarding their changeability.

**Conclusions:**

This study identified general and stage-specific factors relevant for the introduction of physical activity interventions in primary health care. It emphasizes the importance of taking these factors into account when designing introduction strategies, and of giving special attention to the distinct stages of the process. Due to lack of consensus on the changeability of most important factors, the extent to which these factors can be influenced by introduction strategies remains unclear.

## Background

In the last decades many interventions to promote physical activity (PA) in primary health care (PHC) have been proven to be effective in research settings [1-3]. However, within PHC practice, rates of PA promotion are suboptimal [4,5] and interventions are often not delivered as intended by the intervention developers [6-9].

To have an impact on public health, efficacious PHC-based PA interventions need to be effectively introduced in practice. This process involves several stages which often require changes in organizations and professionals’ behavior. In short, organizations and professionals need to make the decision to work with an intervention (i.e., adoption), deliver it as intended (i.e., implementation), and continue to use it over a longer period of time (i.e., continuation) [9-11]. Furthermore, the process, and each of the stages within it, may be influenced by a multitude of factors related to the innovation, adopting person, patient, social setting, organizational context, and innovation methods and strategies [12-17].

Reviews on the introduction of PA interventions in PHC have identified factors influencing professionals’ PA counseling behavior. Barriers that were often mentioned were lack of time, perceived lack of patient receptiveness, and lack of reimbursement [18,19]. Perceived success and sufficient knowledge and skills were reported as facilitating [19]. Taking the comprehensive perspective of factors related to the innovation, adopting person, patient, social setting, organizational context, and innovation methods and strategies [12-17], Huijg et al. systematically reviewed the literature [20] and interviewed intervention stakeholders [21] on factors influencing the introduction of PA interventions in PHC. Both studies resulted in an extensive list of potential influencing factors with some factors similar to determinants discussed in the literature on the introduction of innovations in health care settings [13,14,22] and other factors being an addition to the previous PA intervention literature.

In concordance with Grol et al. [15] and Fixsen et al. [16], Huijg et al. [21] also found that the influence of factors may vary across the distinct stages of the introduction process (i.e., adoption, implementation, and continuation). Various scholars [15,16] already emphasized the importance of studying these distinct stages and taking their specific determinants into account when designing introduction strategies. However, the relevance of factors for the distinct stages of the introduction of PA interventions in PHC has not been previously studied.

Although an overview of potential influencing factors can be helpful when designing strategies to introduce PA interventions in PHC practice, policy makers, intervention managers, and PHC advisors cannot take into account all of the identified factors in this process. Furthermore, in order to investigate the relationship between factors and PA interventions’ adoption, implementation, and continuation in PHC, it might be helpful to identify most relevant factors and refine the list based on factors importance and changeability [23].

The present paper describes a Delphi study designed to reach consensus among experts on the relevance (i.e., importance and changeability) of these factors. The research questions were: 1. which factors, as identified by a systematic literature review [20] and qualitative study [21] are perceived by experts as most important for the adoption, implementation, and continuation of PA interventions in PHC, and 2. how changeable are these factors according to experts?

## Methods

**Figure 1:**
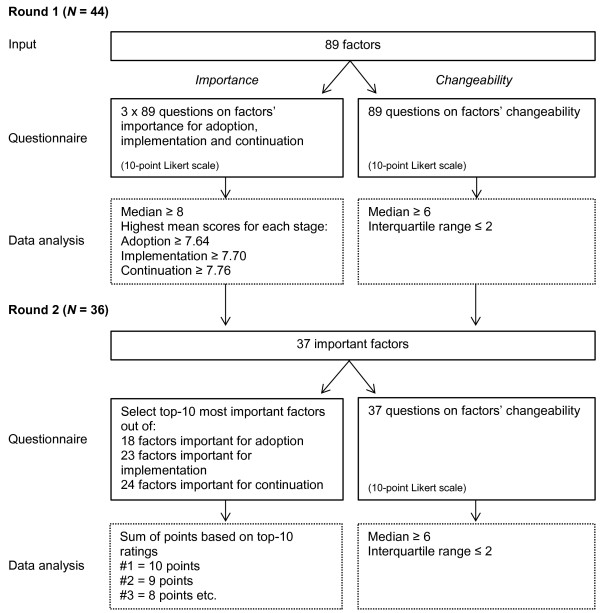
Flow diagram of methods.

A two-round Delphi study was conducted through the Internet by the use of Qualtrics software, version 45433 [24] and within a 4-month time frame (July–October 2011). A flow diagram of the methods is shown in Figure 1.

The Delphi method is a systematic approach that can be used to derive consensus among experts on a topic where scientific knowledge is scarce [25]. Its main characteristics, i.e., anonymity of experts, iteration, controlled feedback, and statistical group response, allow participants to give their opinion freely, change it after having received feedback, and assure that the opinion of every expert is equally represented in the results [25,26].

### First round

#### Procedures and participants

The first round was conducted to facilitate consensus among experts on the importance of factors for the specific stages of the introduction process, i.e., adoption, implementation, and continuation, and on their changeability. Therefore, a variety of people with research and/or practice experience in the field of the introduction of PA interventions in PHC was recruited via research and practice networks (e.g., participants of the qualitative study, LinkedIn groups) and invited to participate by email and telephone. Participating experts were sent an email including the link to the first questionnaire. After two weeks, four weeks, and five weeks, non-respondents received a reminder. In total, 44 experts (response rate of 65%) completed the questionnaire. Completing the questionnaire indicated consent, so no separate consent from participants was obtained. All experts were Dutch and had experience with the introduction of PA interventions in PHC within the following functions: researcher (*n* = 12), policy maker (*n* = 7), intervention manager (*n* = 4), PHC advisor (*n* = 12), and PHC professional (*n* = 9).

#### Questionnaire

The questionnaire consisted of two parts. Part one encompassed 267 structured questions (89 factors × 3 stages) on factors’ importance. Questions were based on the factors identified in the systematic literature review [20] and qualitative study [21] (see Additional file 1) and divided into six categories of factors that may influence the introduction process, i.e., innovation, socio-political context, organization, patient, adopting person, and innovation strategy [12,17]. The experts were asked to rate on a 10-point Likert scale (1 = not at all important, 10 = essential) the importance of each factor for, respectively, the adoption, implementation, and continuation of PA interventions in PHC. For each category of factors an open-ended question was added on whether factors were missing in the list. Part two included 89 structured questions on factors’ changeability. The experts were asked to rate on a 10-point Likert scale (1 = no influence at all, 10 = a lot of influence) the amount of influence they had on each factor during their involvement in the introduction of PA interventions in PHC. Piloting of the questionnaire among health promotion researchers and employees of health promotion institutes indicated that the questionnaire was well received.

#### Data analysis

Median scores were calculated as indicators of factors’ importance for each stage of the introduction process. In concordance with van Stralen et al. [27] factors with a median score of 8 or higher were considered important. Based on median scores, many factors were found to be important. To avoid burdening experts with too many items to decide on their top-10s in the second-round questionnaire, mean scores were calculated to identify *most important factors* for each stage. Based on stages’ grand mean scores of important factors, most important factors were factors with a median score of 8 or higher *and* a mean score of 7.64 or higher for the adoption stage, a mean score of 7.70 or higher for the implementation stage, and mean score of 7.76 or higher for the continuation stage.

Median scores were also calculated for factors’ changeability. Factors were indicated as changeable if they scored a median of 6 or higher. This cut-off value was chosen to be able to include all factors that are considered to be at least somewhat changeable. The interquartile range (IQR) scores were calculated to assess the extent of agreement between the experts on the changeability of each factor [26]. The IQR represents the distance between the 25^th^ and 75^th^ percentile values, with smaller values indicating higher degree of consensus. An IQR score of 1 means that 50% of all the scores given by experts fall within one point on the scale. According to Linstone and Turoff [25] an IQR of 2 or smaller can be considered as good consensus on a 10-point Likert scale.

Differences between expert groups (i.e., researchers, policy makers, intervention managers, PHC advisors, and PHC professionals) with regard to their ratings of factors’ importance and changeability were explored with one-way independent ANOVAs. IBM SPSS Statistics version 19.0 [28] was used for the analyses. The qualitative data on potentially missing factors were scored as ‘new’ or ‘already in the list’.

### Second round

#### Procedures and participants

All experts who completed the first-round questionnaire (*N* = 44) were sent an invitation by email to participate in the second round including the link to the second questionnaire. After one week and two weeks, non-respondents received a reminder. In total, 37 experts (response rate 84%) completed the questionnaire. Of them, 11 were researchers, six were policy makers, three were intervention managers, nine were PHC advisors, and eight were PHC professionals.

#### Questionnaire

The second round was conducted to identify the top-10 most important factors for the specific stages (i.e., adoption, implementation, and continuation) of the introduction process, and their changeability. The questionnaire included the factors that were scored as most important by the experts in the first round (median ≥ 8 and mean ≥ 7.64 for the adoption stage; median ≥ 8 and mean ≥ 7.70 for the implementation stage; median ≥ 8 and mean ≥ 7.76 for the continuation stage). This resulted in a list of 18 factors for the adoption stage, 23 factors for the implementation stage, and 24 factors for the continuation stage; in total 37 different factors (see Table 1). For each stage, the experts were asked to indicate their top-10 of most important factors. Again, open-ended questions were added on whether any factors were missing. For the same set of factors, experts were asked to rate their changeability on a 10-point Likert scale (1 = not changeable at all, 10 = very changeable). In contrast to the first questionnaire, which concerned their own personal influence, experts were asked to rate factors’ changeability in general. This alteration was made because we felt that the group of experts was too heterogeneous for consensus to occur if their own personal influence was taken into account. Again, piloting indicated that the questionnaire was well received.

**Table 1:** Most important factors, stages, and changeability (including consensus)

**Round 1**		**Round 2**
	**Stage**	**Stage & changeability**
**Factors related to the innovation**		
Sustainability	A & C	Continuation
Time investment	Implementation	
Financial feasibility for PHC organizations and professionals	A, I, & C	A, I, & C^C^
Accessibility to the target group	A, I, & C	A, I, & C
Fit with PHC organizations’ and professionals’ objectives	Adoption	
Possibility to tailor intervention to participants’ needs	Implementation	
Complexity of organization intervention	Continuation	
Evidence for intervention effectiveness	Continuation	
**Factors related to the socio-political context**		
Presence of a public health problem	Adoption	Adoption
Support for intervention from government	A, I, & C	
Support for intervention from insurance companies	A, I, & C	Adoption^C^
Support for intervention from local authorities	I & C	
Support for intervention from PHC professionals	A, I, & C	
Presence of intervention champions within community	Adoption	
Availability of PA or sport facilities within community	Continuation	
Network between intervention developer and external parties	Continuation	
Network between PHC and local PA or sport facilities	Continuation	Continuation^C^
**Factors related to the organization**		
Time to deliver the intervention	A, I, & C	A, I, & C
Presence of the target group within the organization	I & C	I & C
Support for intervention from management	Adoption	
Support for intervention from professionals within the organization	A & I	Adoption^C^
Presence of intervention champions within the organization	Adoption	Adoption^C^
**Factors related to the patient**		
Participants’ feedback	I & C	I & C^C^
Relationship between provider and participant	Continuation	
Potential participants’ enthusiasm	I & C	
**Factors related to the adopting person**		
Provider knowledge	Implementation	Implementation^C^
Provider skills	I & C	Implementation^C^
Provider motivation to deliver the intervention	I & C	I & C^C^
Provider attitudes towards PA	Adoption	Adoption^C^
Provider attitudes towards the intervention	A & I	Adoption^C^
Provider attitudes towards intervention effectiveness	A, I & C	Adoption ^C^
Provider experience with intervention effectiveness	I & C	Implementation
**Factors related to the innovation strategy**		
Introduction’s success	I & C	I & C
Time to introduce intervention	A & I	
Intervention materials (participants)	Implementation	
Availability of list of local PA or sport facilities	Continuation	Continuation^C^
Financial resources for introduction	A, I, & C	

*Note*. *A* adoption; *I* implementation; *C* continuation; ^*C*^consensus on changeability + changeable.

#### Data analysis

For changeability, again, the median scores and IQR scores were calculated. Importance was calculated based on the sum of points allocated to the factors based on the experts’ top-10 ranking. For each expert, factors ranked first in the top-10 were allocated ten points, factors ranked second were allocated nine points, and so on. When a factor was not assigned to a top-10, it did not get any points. Differences between expert groups (i.e., researchers, policy makers, intervention managers, PHC advisors, and PHC professionals) with regard to their top-10 rankings and ratings of factors’ changeability were explored with one-way independent ANOVAs. The qualitative data on potentially missing factors were scored as ‘a factor not in the list’ or ‘in depth information on top-10’.

### Ethics

The Medical Ethics Committee of the Leiden University Medical Centre had granted ethical approval of this study (reference number NV/CME 09/081).

## Results

The items and results are shown in Additional file 1. Table 1 shows the most important factors for the different stages of the introduction process and their changeability.

### First round

Experts rated 41 factors as important for the adoption stage (median ≥ 8; M = 7.64), 50 factors as important to the implementation stage (median ≥ 8; M = 7.70), and 56 factors as important to the continuation stage (median ≥ 8; M = 7.76). Intervention’s financial feasibility for PHC organizations and professionals and support for the intervention from insurance companies had the highest median scores regarding all stages (median ≥ 8.5). In addition, related to the continuation stage, several other factors had a median above 8.5: intervention’s accessibility to the target group, evidence for intervention effectiveness, network between PHC and local PA or sport facilities, participants’ feedback, time to deliver the intervention, provider skills, attitudes towards intervention effectiveness, experience with the intervention’s effectiveness, and financial resources for the introduction. The lowest importance ratings (median ≤ 5) were given to delivering the intervention being a fulltime job, competition between PA interventions, routine intervention delivery, and coordination of the intervention in one place. *Most important factors* that were included in the second round questionnaire (median ≥ 8 and mean ≥ 7.64 for the adoption stage; median ≥ 8 and mean ≥ 7.70 for the implementation stage; median ≥ 8 and mean ≥ 7.76 for the continuation stage) are shown in Table 1. Two out of 89 factors were found to be changeable by the majority of experts (median ≥ 6; IQR ≤ 2): provider knowledge and provider attitudes towards the intervention’s effectiveness.

Exploratory one-way independent ANOVAs suggested that the groups of experts differed from one another primarily with regard to how they rated factors’ changeability; significant differences between the groups on changeability ratings were found for around half of the factors. Furthermore, significant differences between the groups were found for 21 out of 267 importance ratings. Groups of experts differed mostly from one another with regard to how they rated factors’ importance for the adoption stage (i.e., 16 out of 21 ratings). In general, PHC professionals rated factors more important and changeable compared to other experts. When experts replied to the open-ended questions on possible missing factors, they provided no ‘new’ factors, but gave a more detailed description of factors already in the list or commented on the complexity of the introduction of PA interventions in PHC.

### Second round

With regard to the top-10s of most important factors, intervention’s financial feasibility, intervention’s accessibility to the target group, and time to deliver the intervention were rated as most important factors for all three stages. Other factors indicated as most important for the adoption stage were: presence of a public health problem, support for the intervention from insurance companies, support for intervention from professionals within the organization, presence of intervention champions within the organization, and provider attitudes towards PA, the intervention, and its effectiveness. Other factors important to the implementation stage were: participants’ feedback, presence of the target group within the organization, provider knowledge, skills, motivation to deliver the intervention, and experience with the intervention’s effectiveness, and introduction’s success. For the continuation stage additional important factors were: intervention’s sustainability, network between PHC and local PA or sport facilities, participants’ feedback, presence of the target group within the organization, provider motivation to deliver the intervention, introduction’s success, and availability of a list of local PA or sport facilities.

From the 37 factors identified as most important from the first round, there was consensus on the changeability of 24 factors (IQR ≤ 2). Among these factors, 23 factors were indicated as changeable (median ≥ 6) and one factor was perceived as unchangeable (i.e., financial resources for the introduction process). With regard to the top-10 most important factors for the distinct stages of the process, there was consensus on the changeability of more than half of the factors. From the three factors important for all three stages, only intervention’s financial feasibility was rated as changeable by the majority of experts (median = 6; IQR = 2). There was no consensus on the changeability of intervention’s accessibility to the target group and time to deliver the intervention. Except for presence of a public health problem, all other most important factors for the adoption stage were considered changeable by the majority of experts. For the most important factors for the implementation stage, participants’ feedback, provider knowledge, skills, and motivation to deliver the intervention were commonly perceived as changeable. There was no consensus on the changeability of the other three factors. For the factors in the top-10 of most important factors for the continuation stage, there was consensus on the changeability of network between PHC and local PA or sport facilities, participants’ feedback, provider motivation to deliver the intervention, and availability of a list of local PA or sport facilities. There was no consensus on the changeability of the other three factors.

Exploratory one-way independent ANOVAs suggested that the groups of experts differed on how they ranked four out of 37 factors and on how they rated the changeability of eight out of 37 factors. In general, PHC professionals rated factors more changeable compared to other experts. Similarly to the first-round questionnaire, experts did not indicate factors were missing in the list.

## Discussion

The objective of this study was to identify factors most relevant for the adoption, implementation, and continuation of PA interventions in PHC by examining experts’ opinions on the importance and changeability of an extensive set of potentially influencing factors based on previous research [20,21].

Factors related to time and money, i.e., time to deliver the intervention within the organization, intervention’s financial feasibility for PHC organizations and professionals, and intervention’s accessibility to the target group, which is most optimal when the intervention is free-of-charge, were found to be important to all three stages. This is not such an unexpected finding, since time and money are important factors in any kind of process and they are frequently mentioned in the leading theoretical models on the introduction of innovations in health care [12,13] and empirical studies [18,20,21]. With regard to the changeability of these factors, there was only consensus on intervention’s financial feasibility, which was rated as changeable, and thus a potentially relevant factor to take into account when introducing PA interventions in PHC. Experts’ rating of intervention’s financial feasibility as changeable and availability of financial resources for the introduction process as unchangeable, might be explained by the fact that financial resources for the introduction process are often dependent on external funding, whereas intervention’s financial feasibility (i.e., the balance between time investment and reimbursement) is mostly within the intervention developers’ own control.

In line with Grol et al. [15] and Fixsen et al. [16] who suggested that different factors may be of critical importance within the distinct stages of the introduction process, the majority of factors were found to be stage specific. With regard to PHC organizations’ and professionals’ *adoption* of PA interventions, results suggest that it is important that there is a public health problem that can be solved by delivering PA interventions and that interventions obtain socio-political support. Furthermore, PHC professionals’ support for the intervention is important for adoption, which is also illustrated by the importance of professionals’ positive attitudes towards PA, the intervention, and the intervention’s effectiveness in this stage. In addition, intervention champions were found to facilitate PHC organizations’ and professionals’ decision to start working with an intervention. The importance of political and financial support for the adoption process has been previously described by Fixsen et al. [16] and is associated with the presence of a public health problem. Furthermore, the importance of the presence of intervention champions within the adoption stage has been confirmed by Carlfjord et al. [29] and Huijg et al. [21] and might reflect the idea that the adoption of PA interventions requires some degree of awareness [16]. Support for the intervention from professionals within PHC organizations can be seen as an important social influence during the adoption process, which together with provider attitudes is a key construct in behavior change theory [30,31]. Except for presence of a public health problem, all factors rated as most important for the adoption stage were found to be changeable, and are thus relevant when designing introduction strategies. For example, attitudes might be changed by arguments and direct experience [23] and intervention champions can be identified and given more emphasis. For the *implementation* of PA interventions it appears to be important that PHC professionals are capable (i.e., have sufficient knowledge and skills) to deliver the intervention and that they experience the intervention’s effectiveness. These results are in line with Bartholomew et al. [23] who state that behavioral capability, skills, self-efficacy, and reinforcement become more important when evolving from the adoption to the implementation of health promotion interventions. Only capability was found to changeable, which can be targeted by the provision of a workshop, which increased PA promotion in previous studies [32-35]. Factors specifically important for the *continuation* stage were the intervention’s sustainability and factors related to participants’ maintenance of PA within the community, i.e., the presence of a network between PHC and local PA or sport facilities, and availability of a list of local PA or sport facilities. Experts agreed that the latter two factors may be targeted in innovation strategies to facilitate long term delivery of PA interventions. The presence of the target group within the organization and the introduction’s success both facilitate the *implementation* and *continuation* of PA interventions in PHC. Furthermore, providers must be motivated and receive participants’ feedback to deliver the intervention in the right way and for a longer period of time, which were found to be changeable following the majority of experts.

Although based on exploratory analysis and no final conclusions can be drawn due to the small sample sizes for all groups, the findings suggest that experts vary with regard to how they rate factors’ importance and changeability depending on the function they have. In general, PHC professionals rated factors more important and changeable than other experts. Differences in changeability ratings might be explained by the fact that experts were asked to rate their personal influence on factors, which is likely to be influenced by the experts’ function. Indeed, in the second round, when general (and not personal) changeability of factors was assessed, a decrease in differences between the expert groups was found.

Some limitations of the study should be noted here. First, by using factors identified through a systematic literature review and a qualitative study as a basis for the first-round questionnaire, we adapted the traditional Delphi method, which usually begins with an open-ended questionnaire to explore experts’ opinions. Advantages of this modification are that it reduces experts’ workload and that the study has a solid ground in previous empirical work [36]. Disadvantages might be that experts do not recognize the factors, since they have not forwarded these themselves, and that they perceive factors missing in the predesigned questionnaire. However, the latter appeared not the case from our analysis of the response to the open-ended questions. Second, only factors with the highest mean scores on importance were included in the second questionnaire, instead of including all factors rated as important (i.e., median scores ≥ 8). This method of selecting factors was chosen to avoid burdening experts with too many items to decide on their top-10s or with another questionnaire round. Third, using the top-10 ranking of factors as a cut-off point to define most important factors for each stage might be an arbitrary choice, since factors rated 11^th^, 12^th^, and so on, could also be important for the introduction process. On the other hand, the method of the study allowed for prioritization of factors. For instance, support for the intervention from insurance companies was rated as important for all three stages in the first round, whereas the results of the second round indicated that this factor was perceived as specifically important for the adoption stage. Fourth, in the first round, where experts rated their personal influence on factors, the lack of consensus might be explained by experts’ different experiences with the introduction of PA interventions in PHC. Although rates on general changeability increased consensus, round two was insufficient in reaching consensus on all factors’ changeability.

## Conclusions

To our knowledge, this was the first study that identified general and stage-specific factors relevant and most important for the adoption, implementation, and continuation of PA interventions in PHC. The results confirm the importance of taking into account the distinct stages and their specific determinants when designing introduction strategies as previously suggested by Grol et al. [15] and Fixsen et al. [16]. Knowledge on which factors are most important for the distinct stages and how changeable they are, can inform policy makers, intervention managers, and PHC advisors in the development of successful introduction strategies. Since consensus could not be reached on the changeability of all most important factors, the extent to which these factors can be influenced by introduction strategies needs further investigation. Finally, researchers can use this explorative study as a basis to further investigate the relationship between these potentially important factors and PHC organizations’ and professionals’ decisions to work with PA interventions, the way they deliver them to the target group, and the continuation of PA interventions in PHC over a longer period of time.

## Abbreviations

PA: Physical activity; PHC: Primary health care.

## Competing interests

The authors declare that they have no competing interests.

## Authors’ contributions

JMH was involved in the design of the study, recruited the participants, collected, analyzed and interpreted the data, and wrote the initial and subsequent drafts of the manuscript. WAG and MRC were involved in the conception and the design of the study, assisted with analyzing and interpretation of the data, and critically revised the manuscript. MWV, NZ, and BJCM were involved in the conception and the design of the study, assisted with analyzing and interpretation of the data, and commented on the manuscript. All authors read and approved the final manuscript.

## Pre-publication history

The pre-publication history for this paper can be accessed here:

http://www.biomedcentral.com/1471-2296/14/142/prepub

## Supplementary Material

Additional file 1 Items and results. Click here for file
