# Supplementary material for: Factors influencing the adoption, implementation, and continuation of physical activity interventions in primary health care: a Delphi study
Source: BMC Fam Pract. 2013 Sep 26;14:142. doi: 10.1186/1471-2296-14-142 (PMC3848920; doi:10.1186/1471-2296-14-142)
Supplement: Additional file 1 — Items and results. [file 1471-2296-14-142-S1.pdf]

Additional file

Additional file 1 – Items and results

	Round 1								Round 2				
	Importance						Changeability		Importance			Changeability	
	A		I		C				A	I	C		
	Mdn	M (≥7.64)	Mdn	M (≥7.70)	Mdn	M (≥7.76)	Mdn	IQR	Sum of points			Mdn	IQR
**Factors related to the innovation**													
Evidence-base	7	6.77	7	6.23	7	6.43	7	4	-	-	-	-	-
Extent to which intervention is known	8	7.20	7	6.61	7	6.50	7	3	-	-	-	-	-
Sustainability	8	**8.05**	8	7.61	8	**8.18**	6	4	99	-	104	5	4
Time investment	8	7.27	8	**7.77**	8	7.64	5	5	-	73	-	6	2
Financial feasibility for PHC organizations and professionals	8.5	**8.45**	9	**8.52**	9.5	**8.98**	5	5	186	141	157	6	2
Intervention's accessibility to the target group	8	**7.75**	8	**8.20**	8.5	**8.36**	5	4	158	123	113	5	3
Delivering intervention is fulltime job	5	5.18	5	5.32	6	5.61	4	6	-	-	-	-	-
Balance between tasks	7	7.00	8	7.36	8	7.73	5.5	5	-	-	-	-	-
Fit with socio-political context	7	6.95	7	7.27	8	7.48	6	5	-	-	-	-	-
Fit with organizational logistics	7	6.66	7	6.82	7	7.05	6	5	-	-	-	-	-
Fit with PHC organizations' and professionals' objectives	8	**7.68**	7	7.05	7	7.16	6	3	82	-	-	7	2
Fit with PHC professionals' knowledge	7	7.09	8	7.43	8	7.32	7	4	-	-	-	-	-
Fit with PHC professionals' routines	7	6.66	7	6.98	7	7.36	6	3	-	-	-	-	-
Relative advantages for socio-political context	7	6.73	7	6.55	7	7.25	6	4	-	-	-	-	-
Relative advantages for PHC organizations	7	7.16	7.5	6.93	7	7.14	5	4	-	-	-	-	-
Relative advantages for PHC	8	7.30	7.5	7.07	8	7.52	5	4	-	-	-	-	-

professionals													
Possibility to tailor intervention to participants’ needs	8	7.34	8	**7.86**	8	7.70	7	4	-	80	-	7	2
Possibility to tailor to PHC organizations’ and professionals’ needs	8	7.41	8	7.52	8	7.55	6	5	-	-	-	-	-
Complexity of organization intervention	7.5	7.36	8	7.64	8	**7.86**	6	4	-	-	87	7	2
Evidence for intervention effectiveness	8	7.48	8	7.30	8.5	**7.91**	5	5	-	-	35	5	3
**Factors related to the socio-political context**													
Presence of a public health problem	8	**8.09**	7	6.98	7	6.68	2.5	5	113	-	-	3	4
Media attention	7.5	7.09	6.5	6.18	6	5.93	5	4	-	-	-	-	-
Socio-political medical culture (preventive)	7	6.77	7.5	7.00	7	6.80	4.5	4	-	-	-	-	-
Formal education on PA and interventions	7	7.05	7	6.75	7	6.30	5	5	-	-	-	-	-
Support for intervention from government	8	**8.14**	8	**7.70**	8	**7.77**	4.5	5	65	22	52	5	3
Support for intervention from insurance companies	9	**8.59**	9	**8.73**	9	**8.66**	5	4	107	67	85	6	2
Support for intervention from local authorities	8	7.61	8	**7.84**	8	**8.07**	5	4	-	61	65	6	1
Support for intervention from PHC professionals	8	**7.84**	8	**7.75**	8	**8.09**	7	3	77	34	27	7	2
Support for intervention from PHC organizations	8	7.45	8	7.50	8	7.59	6.5	4	-	-	-	-	-
Presence of intervention champions within community	8	**8.16**	7.5	7.34	7	7.07	6	4	50	-	-	6	3
Availability of PA or sport facilities within community	6	5.91	7	7.25	8	**7.84**	3.5	6	-	-	90	7	3
Network between intervention developer and external parties	7	7.18	8	7.52	8	**7.89**	7	4	-	-	59	7	2
Network between intervention developer and PHC organizations and professionals	7	7.36	7	7.41	7	7.36	7	3	-	-	-	-	-
Network between intervention	7	7.00	8	7.34	8	7.59	6	5	-	-	-	-	-

providers													
Network between PHC and local PA or sport facilities	7	6.39	8	7.36	9	**8.00**	6	4	-	-	129	7	2
Collaboration between intervention developer and intervention partners	7.5	7.34	8	7.52	8	7.61	7	3	-	-	-	-	-
Collaboration between different PA interventions	5.5	5.52	6	5.50	7	6.45	6	4	-	-	-	-	-
Collaboration between intervention providers	7	6.57	8	7.14	8	7.39	6	4	-	-	-	-	-
Socio-political medical culture (curative)	8	7.55	8	7.25	8	7.36	3.5	4	-	-	-	-	-
Competition between PA interventions	6	6.09	5.5	5.61	5	5.52	3	3	-	-	-	-	-
**Factors related to the organization**													
Time to deliver the intervention	8	**7.86**	8	**8.39**	9	**8.64**	4	6	120	148	92	6	4
Presence of the target group within organization	8	7.50	8	**7.91**	8	**7.82**	3	5	-	114	120	5	4
Support for intervention from management	8	**7.70**	8	7.50	8	7.57	5	6	82	-	-	7	2
Routine of intervention tasks within organization	7	6.50	8	7.36	8	7.45	5	4	-	-	-	-	-
Support for intervention from professionals within organization	8	**7.86**	8	**7.82**	8	7.75	7	4	164	56	-	7	2
Presence of intervention champions within organization	8	**8.00**	7	7.30	7	6.82	6	5	162	-	-	6	2
Collaboration within organization	7	6.98	8	7.55	8	7.59	6	5	-	-	-	-	-
Presence of PA or sport facilities within organization	6	6.20	7	6.61	7	6.45	4	5	-	-	-	-	-
Organizational medical culture (curative)	8	7.34	7	7.00	7	6.98	4	5	-	-	-	-	-
**Factors related to the patient**													
Participants' feedback	8	7.18	8	**8.07**	9	**8.48**	5	5	-	105	93	7	2
Relationship between provider and participant	7	6.82	8	7.61	8	**7.86**	4.5	6	-	-	66	7	2
Potential participants' enthusiasm	7	7.07	8	**7.98**	8	**8.05**	5	5	-	53	21	7	3
Potential participants have other	7	6.59	7	6.52	7	6.66	5	5	-	-	-	-	-

aims for consultation													
Potential participants' medical culture (curative)	7	6.93	6.5	6.66	6.5	6.75	4	5	-	-	-	-	-
**Factors related to the adopting person**													
Knowledge	7	7.14	8	**7.80**	8	7.45	7	2	-	82	-	8	0
Skills	7	7.25	8	**8.11**	9	**8.05**	7	3	-	166	53	8	1
Experience with PA intervention	6.5	6.00	6	6.20	7	6.55	4	4	-	-	-	-	-
Experience with target group	7	6.64	8	7.43	7	7.02	4	6	-	-	-	-	-
Motivation to deliver intervention	8	7.45	8	**8.14**	8	**8.16**	7	3	-	171	94	7	2
Self-efficacy	7	7.14	8	7.43	8	7.36	6	5	-	-	-	-	-
Perception that he or she plays an important role in the intervention's effectiveness	8	7.48	8	7.55	8	7.68	7	3	-	-	-	-	-
Attitudes towards PA	8	**7.73**	8	7.32	7.5	7.20	6	5	158	-	-	7	2
Attitudes towards the intervention	8	**7.70**	8	**7.82**	8	7.73	7	5	131	67	-	7	2
Attitudes towards prevention	8	7.48	7.5	7.09	8	7.14	6	4	-	-	-	-	-
Perception on role or responsibilities with regard to prevention and PA interventions	8	7.43	7	7.18	7	7.14	6	3	-	-	-	-	-
Attitudes towards intervention effectiveness	8	**8.07**	8	**7.95**	9	**8.20**	7	2	151	52	56	8	2
Experience with intervention effectiveness	8	7.09	8	**8.30**	9	**8.75**	7	4	-	119	83	6	3
Priority of intervention delivery	7	6.80	8	7.32	8	7.41	4	4	-	-	-	-	-
Routine of intervention delivery	5	5.73	7	6.70	7	7.09	5	4	-	-	-	-	-
Attitudes towards the target group (motivation)	7	7.00	8	7.27	8	7.45	5	3	-	-	-	-	-
Attitudes towards the target group (maintenance of behavior change)	7	6.66	7	7.09	8	7.30	5	4	-	-	-	-	-
Perception on effect of intervention on relationship with patients	6	5.86	6	6.07	6	5.98	5	4	-	-	-	-	-
**Factors related to the innovation strategy**													
Introduction success	8	7.18	8	**7.84**	8	**8.50**	6	4	-	139	181	6	3
Coordination of intervention in one place	5	5.30	6	5.86	6	5.80	5	5	-	-	-	-	-

Stakeholder involvement in development of intervention	8	7.52	7	7.14	7	7.02	6	4	-	-	-	-	-
Time to introduce intervention	8	**7.73**	8	**7.89**	8	7.32	6	3	70	55	-	7	2
Clarity on tasks	8	7.48	8	7.39	8	6.82	6.5	4	-	-	-	-	-
Clarity on roles	8	7.34	7.5	7.34	7.5	7.07	7	4	-	-	-	-	-
Materials (participants)	7	7.23	8	**7.75**	8	7.20	7	4	-	53	-	8	2
Materials (providers)	7.5	7.18	8	7.41	8	7.20	7	3	-	-	-	-	-
Availability of list of local PA or sport facilities	7	6.77	8	7.45	8	**7.93**	6.5	4	-	-	<u>94</u>	8	2
Reminders	6	5.55	6.5	6.11	7	6.23	5.5	5	-	-	-	-	-
Refinement	6	5.93	7	6.80	8	7.30	7	4	-	-	-	-	-
Research	7	6.84	7	6.45	7	6.95	8	3	-	-	-	-	-
Assistance	8	7.34	8	7.48	7.5	7.18	7	4	-	-	-	-	-
Training	8	7.57	8	7.59	8	7.30	8	5	-	-	-	-	-
Intervision	7	6.80	7	7.20	8	7.30	7.5	3	-	-	-	-	-
Financial resources for introduction	8	**8.02**	8	**8.39**	9	**8.64**	5	5	60	54	79	5	2
Intervention readiness	7	6.59	7	6.86	8	7.27	6	4	-	-	-	-	-

Note. A, adoption; I, implementation; C, continuation; Mdn, median; M, mean; IQR, interquartile range

Bold mean scores indicate most important factors for each stage as scored in the first round, which was based on factors' median score (≥ 8) and mean score (mean ≥ 7.64 for the adoption stage; mean ≥ 7.70 for the implementation stage; mean ≥ 7.76 for the continuation stage). These factors were included in the second round questionnaire.

<u>Underlined</u> sum of points indicate the top-10 of most important factors for each stage as rated in the second round.
